# Genomic Imprinting Variations in the Mouse Type 3 Deiodinase Gene Between Tissues and Brain Regions

**DOI:** 10.1210/me.2014-1210

**Published:** 2014-09-18

**Authors:** M. Elena Martinez, Marika Charalambous, Aabida Saferali, Steven Fiering, Anna K. Naumova, Donald St Germain, Anne C. Ferguson-Smith, Arturo Hernandez

**Affiliations:** Department of Molecular Medicine (M.E.M., D.S.G., A.H.), Maine Medical Center Research Institute, Scarborough, Maine 04074; Centre for Endocrinology (M.C.), William Harvey Research Institute, Barts and The London School of Medicine and Dentistry, Queen Mary University of London, London E1 1BB, United Kingdom; Department of Obstetrics and Gynecology and Human Genetics (A.S., A.K.N.), McGill University, Montréal, Québec, Canada H9X 3V9; Department of Microbiology and Immunology (S.F.), Dartmouth Medical School, Lebanon, New Hampshire 03756; and Department of Genetics (A.C.F.-S.), University of Cambridge, Cambridge CB2 1TN, United Kingdom

## Abstract

The *Dio3* gene, which encodes for the type 3 deiodinase (D3), controls thyroid hormone (TH) availability. The lack of D3 in mice results in tissue overexposure to TH and a broad neuroendocrine phenotype. *Dio3* is an imprinted gene, preferentially expressed from the paternally inherited allele in the mouse fetus. However, heterozygous mice with paternal inheritance of the inactivating *Dio3* mutation exhibit an attenuated phenotype when compared with that of *Dio3* null mice. To investigate this milder phenotype, the allelic expression of *Dio3* was evaluated in different mouse tissues. Preferential allelic expression of *Dio3* from the paternal allele was observed in fetal tissues and neonatal brain regions, whereas the biallelic *Dio3* expression occurred in the developing eye, testes, and cerebellum and in the postnatal brain neocortex, which expresses a larger *Dio3* mRNA transcript. The newborn hypothalamus manifests the highest degree of *Dio3* expression from the paternal allele, compared with other brain regions, and preferential allelic expression of *Dio3* in the brain relaxed in late neonatal life. A methylation analysis of two regulatory regions of the *Dio3* imprinted domain revealed modest but significant differences between tissues, but these did not consistently correlate with the observed patterns of *Dio3* allelic expression. Deletion of the *Dio3* gene and promoter did not result in significant changes in the tissue-specific patterns of *Dio3* allelic expression. These results suggest the existence of unidentified epigenetic determinants of tissue-specific *Dio3* imprinting. The resulting variation in the *Dio3* allelic expression between tissues likely explains the phenotypic variation that results from paternal *Dio3* haploinsufficiency.

The type 3 deiodinase (D3) modulates thyroid hormone (TH) action by converting both the prohormone T_4_ and the active hormone T_3_ into inactive metabolites (1–3). Its high expression in the pregnant uterus, fetus, and placenta (4, 5) and in the developing and adult central nervous system (CNS) (6, 7) suggests that D3 is important for the maintenance of appropriate levels of TH in the fetus and the adult. In this regard, we have shown that mice lacking D3 (D3KO or *Dio3*^−/−^ mice) are overexposed to T_3_ during development and subsequently manifest marked deficits in the maturation and function of the thyroid axis, severe growth retardation, and impaired viability and fertility (8, 9). These mice also exhibit alterations in cardiovascular (10) and sensory functions (11, 12), glucose and insulin homeostasis (13), and brain gene expression patterns (14, 15), demonstrating a critical role for D3 in many pathophysiological outcomes.

We and others have demonstrated that *Dio3*, the gene that codes for D3, is subject to genomic imprinting in the mouse (16, 17). Genomic imprinting is an epigenetic phenomenon that affects a small percentage of mammalian genes (18–20). Imprinted genes are preferentially or exclusively expressed from one of the alleles, depending on the parental origin (18). Although the mechanisms underlying genomic imprinting are not fully understood, a common feature of imprinted loci is the differential methylation (imprint) between parental alleles in key regulatory genomic sequences. These imprints ultimately translate into the allele-specific expression or repression of certain genes within the imprinted domain (18–20). Genomic imprinting results in a high degree of monoallelic gene expression, and thus poses a higher risk of pathologies, as a random mutation in just one copy of the imprinted gene may lead to deleterious loss or gain of function. Furthermore, alterations in the dosage of imprinted genes due to disrupted genomic imprinting result in abnormalities in development and adult physiology (21, 22). In humans, aberrant genomic imprinting is the cause of severe developmental, neurological, and metabolic syndromes (23–27).

*Dio3* belongs to the *Dlk1-Dio3* imprinted domain (28), and in the mouse fetus, it is expressed preferentially from the allele inherited from the father (16, 17). However, the repression of the maternal *Dio3* allele is not complete (17), and in the placenta, considerable expression originates from the maternal allele (29), indicating that *Dio3* imprinting in this tissue is significantly relaxed. We have also reported a lesser degree of genomic imprinting in the heads of fetal mice (17). Taken together, these observations suggest that allelic contributions to *Dio3* expression vary in a tissue-specific pattern, with resultant functional and phenotypic implications. In the context of the milder gross phenotype that we observe in heterozygous mice with a mutated paternal *Dio3* allele when compared with that of null *Dio3* mice, herein we use our previously described D3KO mouse and a second novel model of targeted *Dio3* disruption to analyze *Dio3* allelic expression in various tissues and in regions of the CNS. We show that *Dio3* imprinting varies significantly between tissues and developmental stages, especially in the CNS. This suggests that epigenetic information regulates *Dio3* expression in a tissue- and/or cell-specific manner and that the resultant alterations in the imprinting pattern of *Dio3* effect developmental outcomes.

## Materials and Methods

### Animals, genotyping, and tissue harvesting

Animals were kept under a 12-hour light cycle and provided food and water ad libitum. Animals were killed by asphyxiation with CO_2_ (adults and weanlings) or by decapitation (neonates and fetuses). Mice of both sexes were used together because no differences in the results obtained were appreciated between males and females. For RNA isolation and D3 enzymatic activity, tissues were dissected, immediately frozen on dry ice, and stored at −70°C until further use. Brain regions were identified and harvested according to the mouse atlas by Paxinos and Franklin (30). Animal procedures were approved by the Institutional Animal Care and Use Committees of Dartmouth College and the Maine Medical Center Research Institute. Genotyping of the D3KO animals carrying the triple-point mutation was performed by a PCR of genomic DNA from tail snips as previously described (8). Genotyping of mice carrying the novel deletion of the *Dio3* gene was performed by Southern analysis (see below) or by a double PCR using a pair of primers that amplify a DNA fragment of the native *Dio3* gene and another pair of primers that amplify a DNA fragment specific to the deleted allele (Supplemental Methods).

### Mouse embryonic stem (ES) cells and introduction of mice carrying a deletion of *Dio3*

We (17) described the strategy used to target the *Dio3* gene using standard homologous recombination techniques. We used the R1 ES cell line (31), which originated from the 129/Sv mouse strain. Targeted clones were identified by Southern analysis, injected into C57/BL6 blastocysts and reimplanted in CD1 foster mothers. Chimeric males that showed germline transmission were mated to C57/BL6 females to test for the germline transmission of the mutation. Chimeric males were then mated with 129/Sv females to establish the mutant line in a 129/Sv background.

### D3 activity and serum levels of TH

D3 enzymatic activities were determined as previously described (8). In brief, tissues were homogenized in a 10-mM Tris-HCl, 0.25 sucrose buffer (pH 7.4). A suitable volume of tissue homogenate was used in the enzymatic reaction to ensure that deiodination do not exceed 40% and was proportional to the amount of protein content. Tissue homogenates were incubated at 37°C for an hour with 2 nM ^125^I-labeled T_3_ (PerkinElmer) in the presence of 25 mM dithiothreitol. Deiodination was determined based on the percentage of ^125^I-3,3′-diiodothyronine produced. The latter was determined by measuring the amount of radioactivity associated with the reaction products after separation by paper chromatography as described (32). Total serum thyroid levels were determined using RIA kits following the manufacturer's directions (TKT4 and TKT3; Siemens). Serum TSH was determined by a RIA as previously described (9).

### DNA and RNA isolation and Northern and Southern analysis

Total RNA and poly(A+) RNA were isolated from brain tissues using the RNAeasy kit (QIAGEN), including deoxyribonuclease treatment, and the poly(A+) kit from Ambion (Now Life Technologies). *Dio3* Northern analysis was performed following standard protocols. In brief, total and poly(A+) RNA samples were electrophoresed in a denaturing 1% agarose gel containing formaldehyde and blotted onto a Nytran membrane (GE Life Sciences). The blots were hybridized at 42°C in buffer containing 50% formamide, washed with 0.1× saline sodium citrate/0.1% sodium dodecyl sulfate at 65°C and autoradiographed for 1–7 days. Probes were labeled with radioactive ^32^P-dCTP (MP Biomedicals, Inc) using the oligolabeling kit (Pfizer) and were purified through G-50 columns (Pfizer). A 1.35-kb *Xho*I restriction fragment comprising the *Dio3* coding region and part of the 3′-untranslated region was used as a probe.

DNA was isolated from tail snips using a kit from QIAGEN. A Southern analysis was performed using standard protocols on genomic DNA (gDNA) digested with *Eco*RI. A 0.7-kb *Sac*I/*Eco*RI restriction fragment located immediately outside the 5′ end of the targeting sequencing was used as a probe.

### RT-PCR analysis of allelic expression

Total RNA (1 μg) was reverse transcribed for 1 hour at 42°C with 1 μL of Moloney murine leukemia virus reverse transcriptase (Life Technologies). A reaction without the reverse transcriptase was performed as a negative control to ensure no significant amplification of gDNA in the downstream PCR. The mix was heated at 75°C for 15 minutes to inactivate the reverse transcriptase and diluted 1 to 20 with water. Aliquots of the mixes from a given experiment were pooled before dilution to establish the first point of an internal standard. Three consecutive one to four dilutions of this standard were done to generate three additional standard points. Ten microliters of each of the diluted samples were mixed with 12.5 μL of SYBR Select master mix from Life Technologies and 2.5 μL of the appropriate gene-specific primer mix (3.33 pmol/μL each). The mixture was subjected to PCR cycling using a MyiQ single color real-time PCR detection system from Bio-Rad Laboratories. The PCRs were performed in triplicate, and data for each gene and sample were read from the standard curve. *Gapdh* was used as a control gene, its expression level not varying significantly between experimental groups. The expression levels for each gene are reported in arbitrary units, after correction by *Gapdh* expression. The sequences of the primers used are shown in Supplemental Methods.

### Bisulfite sequencing of the intergenic differentially methylated region (IG-DMR)

The sodium bisulfite sequencing methylation assay was performed as described (33) using 500 ng to 1 μg of DNA from 129/Sv mouse tissues of the neonatal age (P1). Briefly, two independent PCR replicates were performed using bisulfite-treated DNA as template. PCR products were gel purified, combined, and cloned using the TopoTA cloning kit (Invitrogen). Clones were analyzed and sequenced at the McGill University and Genome Québec Innovation Centre sequencing platform. Duplicate clones or clones with incomplete C-to-U conversion were excluded from the analysis. Information about the sequence and CpG groups analyzed can be found in Supplemental Methods.

### Pyrosequencing

Bisulfite conversion and pyrosequencing was carried out according to Sun et al (34) with the following modifications: gDNA (1 μg) was treated using the Imprint DNA modification kit (Sigma) in accordance with the manufacturer's instructions for the two-step conversion and eluted in 20 μL. Primer sequences, annealing temperatures, and cycle number are shown in Supplemental Methods. Pyrosequencing was performed on the PSQ HS96 system using PyroGold Q96 SQA reagents (QIAGEN). The degree of methylation at CpG sites (without distinguishing between maternal and paternal alleles) was determined by pyro-Q CpG software (Biotage). Information about the sequence and CpG groups analyzed can be found in Supplemental Methods.

### Statistics

We used the GraphPad Prism software for statistical analysis. Unless stated otherwise, differences between more than two experimental groups were determined by ANOVA and Tukey's post hoc test, whereas differences between two groups were determined by the Student's *t* test. *P* < .05 was considered statistically significant.

## Results

### Paternal inheritance of the mutated *Dio3* allele leads to a milder gross phenotype than that in null *Dio3* mice

**Figure 1.**
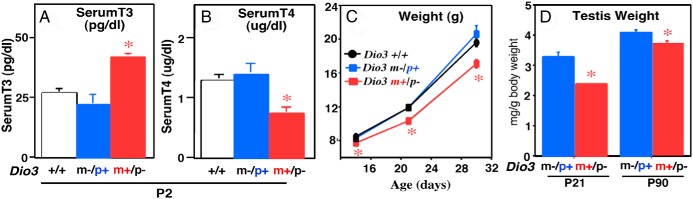
Altered phenotypes in heterozygous mice with paternal inheritance of the mutated *Dio3* allele. A and B, Serum levels of T_3_ and T_4_ at P2. Data represent the mean ± SEM of determinations performed on five to eight serum samples. For each experimental group, each sample is composed of pooled serum from two to three individual mice of both sexes. *, *P* < .01 as determined by ANOVA and Tukey's post hoc test. C, Weight. Data represent the mean ± SEM of weights measured in 8–12 animals per experimental group. Only animals born to litters of five to seven pups in size were included. *, *P* < .01 as determined by ANOVA and Tukey post hoc test. D, Testicular weight relative to body weight. Data represent the mean ± SEM of weights measured in eight, 18, 28, and 22 animals, respectively. *, *P* < .01 as determined by Student's *t* test.

Given that *Dio3* is preferentially expressed from the paternally inherited allele (16, 17), one would predict that heterozygous animals that inherited the mutation from the father (*Dio3* m+/p−) would manifest a phenotype similar to that of a homozygous *Dio3*^−/−^ mutant. Such is at least partially the case; mice with the *Dio3* m+/p− genotype had a significantly milder phenotype than mice that were completely D3 deficient (8). For example, unlike *Dio3*^−/−^ mice, adult *Dio3* m+/p− mice do not exhibit significant changes in their serum levels of thyroid hormones, when compared with the wild types (*Dio3*^+/+^) or with heterozygous animals with maternal inheritance of the mutation (*Dio3* m−/p+ mice) (data not shown) (10). However, an increased serum level of T_3_ and a partially suppressed serum T_4_ level are noted at postnatal day (P) 2 (Figure 1, A and B). At this age, serum TSH trended lower (68.44 ± 10.09 mU/L in *Dio3* m+/p− mice vs 85.85 ± 11.65 mU/L in *Dio3* m−/p+ mice, n = 4 and 5), but the difference was not statistically significant. These thyroid parameters indicate neonatal overexposure to T_3_ and partial thyroid axis suppression in *Dio3* p−/m+ mice. They are also significantly growth retarded and the weight of their testes is reduced (Figure 1, C and D). These abnormalities indicate that reduced *Dio3* dosage has phenotypic consequences. However, these gross phenotypes are significantly milder than those in *Dio3*^−/−^ mice.

Previous observations indicated that P2 *Dio3*^−/−^ mice feature a 5-fold increase in serum T_3_, an 80% reduction in serum T_4_, and a 30% reduction in weight as young adults (8). Testes size in young *Dio3* −/− males is also reduced 60%, when compared with *Dio3*^+/+^ littermates (unpublished observations, Hernandez, A.). Compared with that of *Dio3*^−/−^ mice, the milder phenotype of *Dio3* m+/p− mice suggests that *Dio3* expression from the maternal allele is partially compensating the lack of expression from the paternal allele in these animals.

### *Dio3* is preferentially expressed from one allele in mouse ES cells

**Figure 2.**
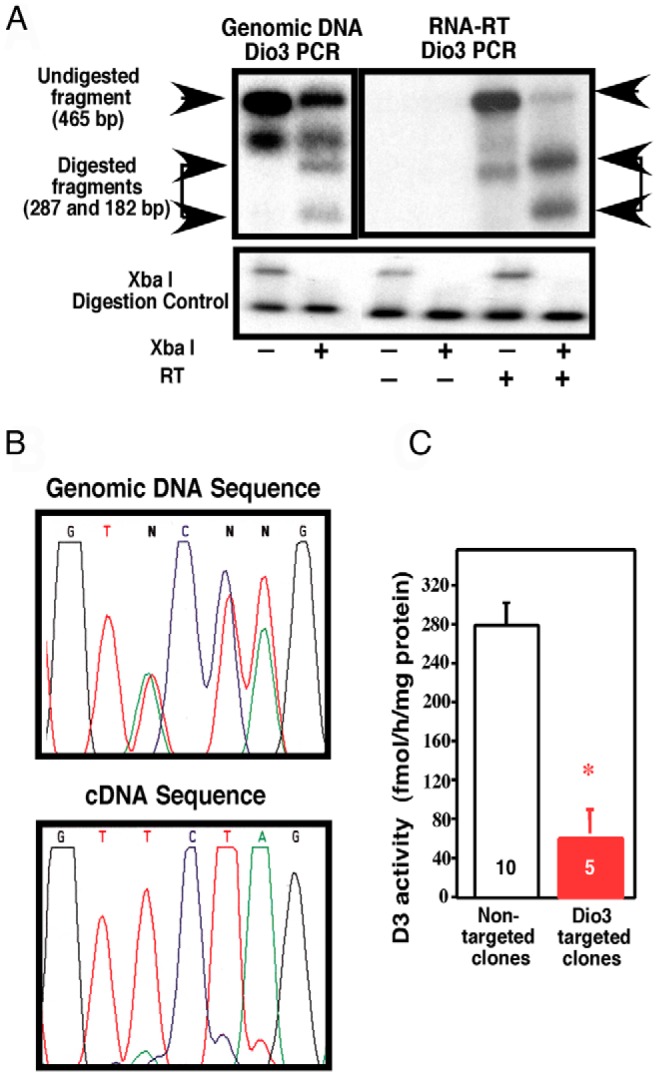
Preferential allelic expression of *Dio3* in mouse ES cells. A, Representative Southern analysis of restriction fragments from gDNA and cDNA of an ES cell clone in which one allele of *Dio3* has a triple-point mutation creating an *Xba*I restriction site. A DNA fragment containing the mutation was amplified by PCR, digested with *Xba*I, and submitted to Southern analysis using a *Dio3* cDNA as a probe. A plasmid that was linearized as a result of *Xba*I activity was included as an internal control of *Xba*I digestion. Initial PCR band and products of *Xba*I digestions are marked by arrows. The band not pointed at by arrows is a DNA heteroduplex PCR artifact not susceptible to *Xba*I digestion. RT, reverse transcriptase. B, Genomic DNA and RNA-derived cDNA sequence of the triple-point mutation in a targeted ES cell clone. C, D3 activity in 5 *Dio3*-targeted clones and 10 nontargeted clones. Data represent the mean ± SEM. *, *P* < .01 as determined by Student's *t* test.

We have previously reported the targeting of the *Dio3* gene in mouse ES cells to generate a mouse deficient in the type 3 deiodinase (17). We have used *Dio3* targeted and nontargeted ES cell clones from that experiment to analyze *Dio3* expression. Targeted clones are heterozygous for a *Dio3* mutation that introduces an *Xba*I restriction site in the selenocysteine codon, thus rendering the enzyme inactive (35). We prepared gDNA and cDNA from *Dio3*-targeted clones and used PCR to amplify a DNA fragment containing the inserted *Dio3* mutation. *Xba*I digestion of these fragments (Figure 2A) revealed partial digestion when the fragment had a gDNA origin, whereas almost complete digestion occurs when the fragment was amplified from cDNA generated by reverse transcription of total RNA. Sequencing of the mutation site in these fragments demonstrated that mutated DNA accounts for approximately half of the gDNA but for a much higher percentage of the RNA-derived cDNA (Figure 2B). Similar results were obtained for all five *Dio3*-targeted clones. In addition, D3 activity in these targeted clones was less than 20% of the average D3 activity measured in nontargeted clones (Figure 2C). These results indicate not only that *Dio3* is preferentially expressed from one of the alleles in mouse ES cells but also that the preferentially expressed allele is targeted more frequently in the homologous recombination event.

### *Dio3* allelic expression in the fetal head

**Figure 3.**
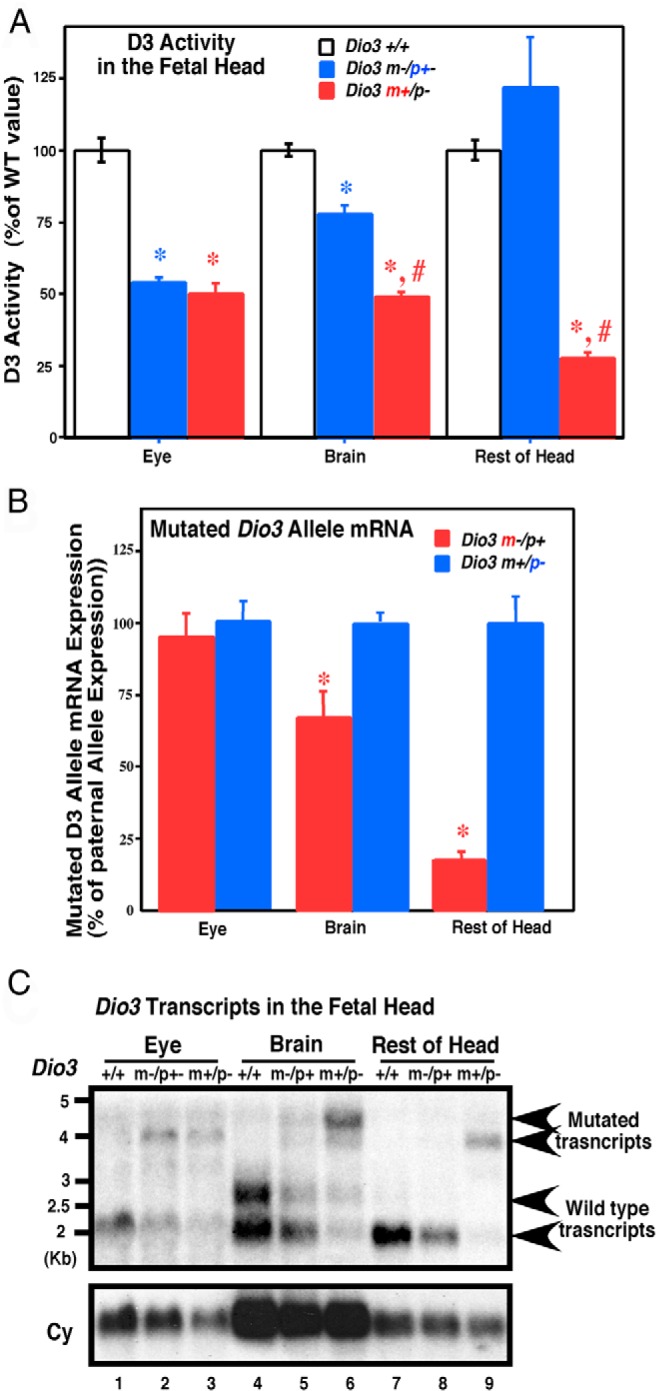
*Dio3* allelic expression varies in the E15.5 fetal head. A, D3 activity in the eye, brain, and the rest of head structures of E15.5 mouse fetuses. Bars represent the mean ± SEM of 36, 14, 22, 38, 14, 27, 14, seven, and 11 individual samples. Data are expressed as a percentage of wild-type values. *, #, *P* < .01 vs *Dio3*^+/+^ and *Dio3* m−/p+, respectively, as determined by ANOVA and Tukey's post hoc test. B, mRNA expression of the mutated allele. Bars represent the mean ± SEM of six determinations, and data are expressed as a percentage of the values for *Dio3* m−/p+ mice. *, *P* < .01 as determined by the Student's *t* test. C, Northern analysis of fetal head RNA. Total RNA (20 μg) was used for eye and head, whereas polyA+ enriched RNA (5 μg) was used for brain. RNA samples were pooled from at least three different animals. Cyclophilin hybridization (Cy) is shown for informative purposes only because the purpose of this experiment was to assess the relative abundance of mutated and wild-type *Dio3* within a specific pooled sample.

Previous data suggested that allelic contribution to *Dio3* expression may be only slightly biased toward the paternal allele in the fetal head (17). We have analyzed the allelic contribution to *Dio3* expression in the fetal eye, brain, and the rest of craniofacial structures. We used animals generated by reciprocal matings of *Dio3*^+/−^ and *Dio3*^+/+^ mice so that we could track the parental origin of the mutated allele in the offspring and thus the allele that is contributing to *Dio3* expression (8). Fetal eye D3 activities in heterozygous mice that inherited the mutation from the father (*Dio3* m+/p−) were similar to that in heterozygous mice that inherited the mutation from the mother (*Dio3* m−/p+), and both were approximately 50% of D3 activity measured in wild-type (*Dio3*^+/+^) mice (Figure 3A). D3 activity in the brain of *Dio3* m−/p+ mice was 20% lower than in *Dio3*^+/+^ mice, whereas it was significantly higher than in *Dio3* m+/p− mice, in which D3 activity was reduced 55% compared with that in wild-type mice. In the tissues comprising the rest of the head and craniofacial structures, D3 activity in *Dio3* m−/p+ mice was not significantly reduced compared with that of wild-type animals, but a 75% reduction was observed in *Dio3* m+/p− mice (Figure 3A), indicating strong preferential expression from the paternal allele in this tissue.

*Dio3* mRNA expression from the mutated allele was evaluated by real time quantitative PCR using primers specific for the mutated *Dio3* allele. Mutated *Dio3* mRNA was expressed at similar levels in the eyes of *Dio3* m−/p+ and *Dio3* m+/p− fetuses (Figure 3B). In the brain, the expression of the mutated *Dio3* mRNA was reduced in *Dio3* m+/p− fetuses compared with that in *Dio3* m−/p+ fetuses. In the rest of the fetal head, this reduction was more marked, indicating a stronger preferential *Dio3* expression from the paternal allele. These activity measurements indicate a strong correlation between *Dio3* mRNA and protein levels and confirm the previously defined imprinting pattern based on mRNA expression levels.

Because the rodent brain expresses *Dio3* transcripts that are larger than the 2.2-kb transcript (7) that is most abundant in placenta and fetal tissues, we also performed a Northern analysis of mRNA from fetal tissues. These samples were extracted from mice in which the neomycin cassette used for targeting in ES cells was still present in the 3′-untranslated region of the *Dio3* mRNA (17). As a result, the *Dio3* mRNA species carrying the mutation is approximately 2 kb larger than the native *Dio3* mRNA. As expected, *Dio3* mRNA transcripts above 4 kb in size are detected in tissues from *Dio3*^+/−^ animals. In the fetal eye and nonneural craniofacial structures of the wild-type mice, the typical 2.2-kb transcript is essentially the only one detected (Figure 3C), whereas a larger, similarly abundant, 2.7-kb *Dio3* transcript is also observed in the wild-type fetal brain (Figure 2C, lanes 1, 4, and 7). The abundance of the mutated 4-kb and the native 2.2-kb *Dio3* transcript is similar in the eye of *Dio3* m+/p− and *Dio3* m−/p+ animals (Figure 3C, lanes 2 and 3), indicating biallelic *Dio3* expression in this tissue. However, in the nonneural head samples, the mutated transcript was much more abundant than the native transcript in *Dio3* m+/p− mice, whereas the native *Dio3* transcript was much more abundant than the mutated one in *Dio3* m−/p+ mice (Figure 3C, lanes 8 and 9), demonstrating strong preferential expression from the paternal allele. Similarly, in the fetal brain Figure 3C, (lanes 5 and 6), transcripts that originated in the paternal allele are also more abundant, although the pattern is not as marked as in the tissues contained in the rest of the head. Overall, these results indicate no imprinting of *Dio3* in the fetal eye and marked preferential *Dio3* expression from the paternal allele in the craniofacial tissue and, to a lesser degree, in the fetal brain.

### *Dio3* allelic expression in the perinatal brain

**Figure 4.**
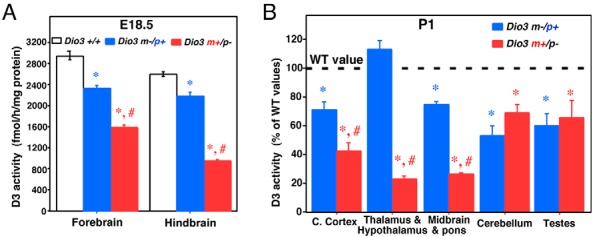
*Dio3* allelic contributions to D3 activity vary in the perinatal brain. A and B, D3 activity in regions of the perinatal brain in *Dio3*^+/+^, *Dio3* m−/p+, and *Dio3* m+/p− mice. Data represent the mean ± SEM of 18, four, and seven determinations (A) or eight determinations (B). *, #, *P* < .01 vs *Dio3*^+/+^ or *Dio3* m−/p+, respectively, as determined by ANOVA and Tukey's post hoc test. WT, wild type.

Based on D3 activity data, brain *Dio3* follows a similar pattern of allelic expression in late gestation. At embryonic day (E) 18.5, *Dio3* expression is reduced in the forebrain and hindbrain of *Dio3* m−/p+ animals compared with that of *Dio3*^+/+^ mice (Figure 4A), but it is reduced significantly further in *Dio3* m+/p−, indicating preferential expression from the paternal allele. At P1, a marked imprinting pattern of expression is observed in the thalamus and hypothalamus, with normal and very reduced *Dio3* expression in *Dio3* m−/p+ and *Dio3* m+/p− animals, respectively (Figure 4B). No imprinted pattern of expression is noticed in the cerebellum, in which *Dio3* expression seems equally biallelic. A relaxed pattern of imprinting is observed in the cerebral cortex and the midbrain and pons. In this case, significant preferential expression from the paternal allele is noted, but it is not sufficient to achieve *Dio3* expression levels comparable with those in wild-type mice (Figure 4B). Lack of *Dio3* imprinting is noted in certain nonneural tissues, such as the newborn testes (Figure 4B).

### *Dio3* allelic expression in the late postnatal brain

**Figure 5.**
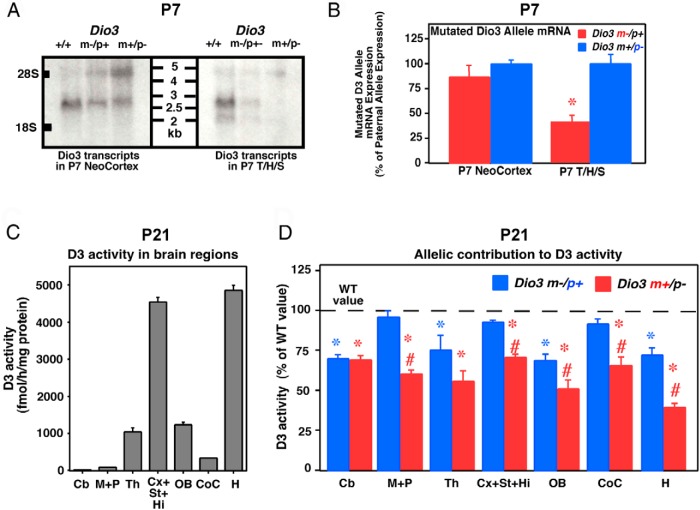
*Dio3* allelic contribution to D3 activity vary in P7 and P21 brain regions. A, Northern blot analysis showing *Dio3* mRNA transcripts in the neocortex and thalamus/hippocampus/striatum (T/H/S) of *Dio3*^+/+^, *Dio3* m−/p+, and *Dio3* m+/p− P7 mice. PolyA+-enriched RNA (5 μg) from four mice per group was used. B, Real-time PCR quantification of the *Dio3* mRNA transcribed from the mutated allele in *Dio3* m−/p+ and *Dio3* m+/p− P7 mice. Data represent the mean ± SEM of five determinations. *, *P* < .01 as determined by the Student's *t* test. C, D3 activity in brain regions of P21 *Dio3*^+/+^ mice. Data represent the mean ± SEM of eight determinations. D, D3 activity in brain regions of *Dio3* m−/p+ and *Dio3* m+/p− P21 mice as compared with wild-type values (dotted line). Cb, cerebellum; CoC, corpus colliculus; Cx+St+Hi, cortex, striatum, and hippocampus; H, hypothalamus; M+P, midbrain and pons; OB, olfactory bulb; Th, thalamus; WT, wild type. Data represent the mean ± SEM of eight determinations. *, #, *P* < .01 vs *Dio3*^+/+^ or *Dio3* m−/p+, respectively, as determined by ANOVA and Tukey's post hoc test.

Northern analysis of P7 brain mRNA reveals that the larger 2.7-kb *Dio3* transcript is the only one detected in the neocortex (Figure 5A, left panel) and does not show significant preferential allelic expression; mutated (and larger because it contains the neomycin cassette) and the wild-type version of this RNA species appear similarly abundant in *Dio3* m−/p+ and *Dio3* m+/p− mice. Quantification of the mutated *Dio3* mRNA species reveals similar levels of expression in heterozygous mice, irrespective of the parental origin of the mutated allele (Figure 5B, left panel). In a P7 specimen comprising the thalamus, hippocampus, and striatum, both 2.2- and 2.7-kb *Dio3* transcripts are detected, the latter being more abundant (Figure 5A, right panel). In this tissue, the pattern of allelic expression in *Dio3* m−/p+ and *Dio3* m+/p− mice still shows significant preferential expression from the paternally inherited allele (Figure 5, A and B, right panel).

At P21, *Dio3* +/+ mice exhibit wide variation in *Dio3* expression levels across brain regions, D3 activity being very high in the hypothalamus and in the cortex, striatum, and hippocampus; low in the cerebellum, midbrain, and pons and in the corpus colliculus; and intermediate in the amount in the olfactory bulb and thalamus (Figure 5C). At this age, thalamic and cerebellar *Dio3* shows no imprinting pattern of expression, D3 activity being comparable in *Dio3* m−/p+ and *Dio3* p−/m+ mice and significantly lower than in *Dio3*^+/+^ mice (Figure 5D). The rest of the brain regions analyzed still exhibit preferential expression from the paternal allele, although the relative contribution of this allele to overall *Dio3* expression appears reduced compared with previous developmental stages. Thus, at this stage, expression from the paternal allele is sufficient to achieve normal levels of *Dio3* expression in the midbrain and pons, corpus colliculus, and the cortex, striatum, and hippocampus, whereas the maternal allele can achieve more than 50% of the normal levels of expression when the paternal allele is mutated (Figure 5D).

### Methylation of the IG-DMR and maternally expressed gene 3 (Meg3) intron in different tissues

**Figure 6.**
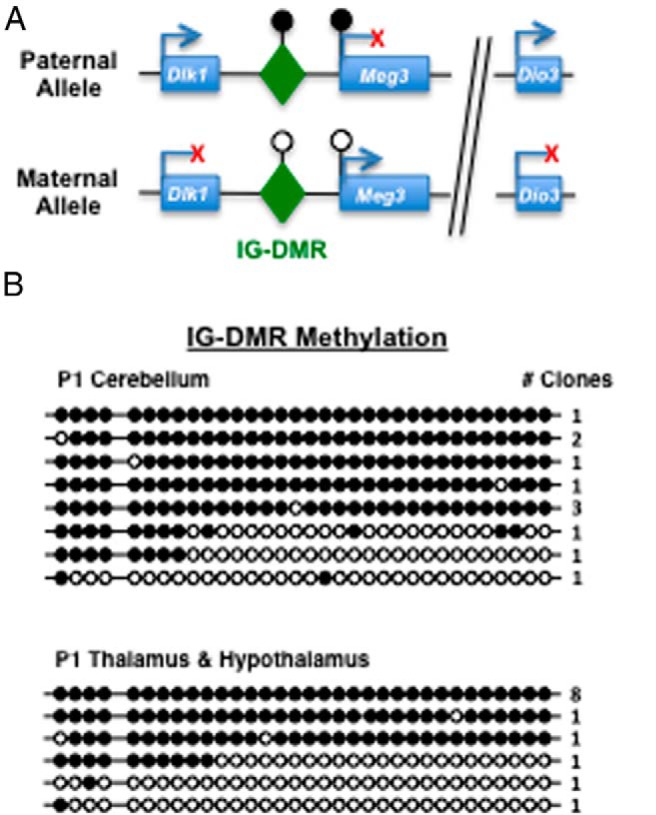
Methylation status of the IG-DMR in the cerebellum and hypothalamus/thalamus of P1 mice. A, Simplified diagram of genomic elements studied in the *Dlk1-Dio3* imprinted domain. B, Bisulfite sequencing methylation assay of 33 CG pairs in DNA isolated from tissues of *Dio3*^+/+^ mice. Closed circles indicate methylated CGs and open circles indicate unmethylated CGs. Each row corresponds to a particular methylation pattern, and the number of clones with each pattern is indicated at the right. Overall methylation in all the clones analyzed is indicated in parentheses for each tissue. Cb, cerebellum; CoC, corpus colliculus; Cx+St+Hi, cortex, striatum, and hippocampus; H, hypothalamus; M+P, midbrain and pons; OB, olfactory bulb; Th, thalamus.

To begin to address the mechanisms of the differences in *Dio3* imprinting between tissues, we analyzed the methylation pattern of the IG-DMR (Figure 6A). This region regulates imprinting in the *Dlk1-Dio3* imprinted domain and is usually methylated in the paternal allele in the mouse fetus (36), in which it appears to play a role in the repression of the maternal copy of *Dio3* (28, 36). First, the methylation status of 33 CpGs corresponding to the IG-DMR (37) was assessed by bisulfite sequencing of DNA isolated from the cerebellum and hypothalamus/thalamus of P1 mice. We could not distinguish between maternal and paternal alleles in these samples, but we observed two types of clones from both tissues, either hypomethylated or hypermethylated (Figure 6B). Overall levels of methylation were comparable in both tissues despite the observation that allelic expression of *Dio3* is different in those tissues, biallelic in the cerebellum and preferentially paternal in the hypothalamus. This suggests that loss of *Dio3* imprinting in the cerebellum is not due to changes in the methylation status of the IG-DMR.

**Figure 7.**
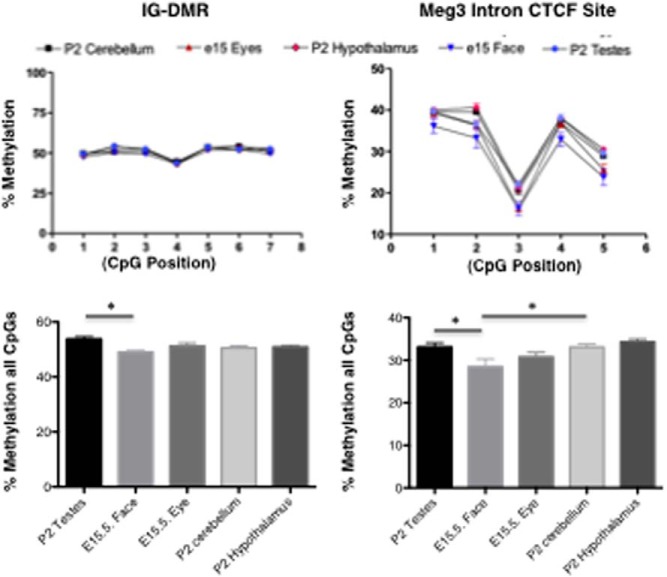
Methylation status of the IG-DMR and *Meg3* intron in various tissues. Methylation percentage of seven and five CpG pairs in the IG-DMR and the *Meg3* intron, respectively, as determined by pyrosequencing on DNA samples isolated from *Dio3*^+/+^ mice. Data represent the mean ± SEM of four determinations. *, *P* < .01 as determined by ANOVA and Tukey's post hoc test.

Pyosequencing analysis of the methylation status of seven CpG (CG5 to CG12) of the IG-DMR indicated approximately 50% methylation in the P2 cerebellum, hypothalamus, and testis and in the fetal eye and face (Figure 7, left panel). Modest but significant differences in the degree of methylation were noticed at P2 between craniofacial structures and the testis.

Using the same technique and the same tissues, we analyzed the methylation status of a region at the 5′-end of the *Meg3* gene (or gene trap locus 2, *Gtl2*), including the first intron. This region is differentially methylated between alleles and contains a putative binding site for the zinc finger protein CCCTC binding factor (CTCF), which plays a role in the regulation of imprinting (28) (Figure 6A). Overall methylation was in the range of 30% for all tissues (Figure 7, right panel). Again, modest but significant differences in methylation level were observed, with fetal craniofacial structures exhibiting significantly lower methylation than the neonatal testes and cerebellum.

In summary, the changes in methylation profiles are associated with differences in *Dio3* allelic expression in some tissues, suggesting an impact on the regulation of *Dio3* genomic imprinting. But this is not the case for all tissues, an observation that points to other tissue-specific mechanisms influencing the epigenetic regulation of *Dio3*.

### Deletion of the *Dio3* gene and promoter does not change imprinting at the *Dio3* locus

**Figure 8.**
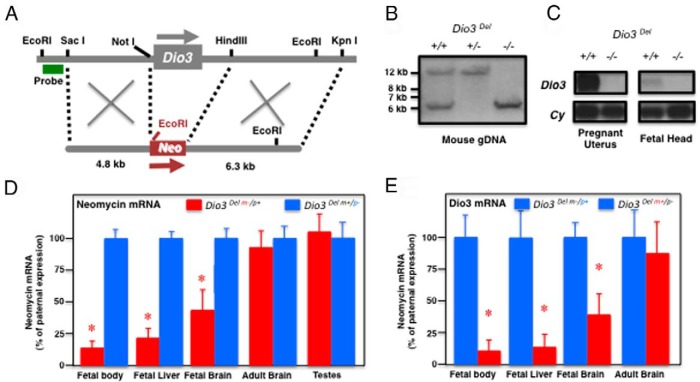
Allelic expression in the *Dio3* locus in the absence of *Dio3* gene and promoter. A, A mouse model was generated featuring an approximately 4.5-kb deletion and replacement with phosphoglycerate kinase-neo that included the *Dio3* gene and promoter (*Dio3*^Del−/−^). B, Southern blot analysis after *Eco*RI digestion of tail snip DNA isolated from *Dio3*^+/+^, *Dio3*^Del+/−^, and *Dio3*^Del−/−^ mice. C, Northern blot analysis of *Dio3* 2.2-kb mRNA transcript in the pregnant uterus and fetal head of *Dio3*^+/+^ and *Dio3*^Del−/−^ mice, demonstrating the absence of *Dio3* mRNA in mice homozygous for the deletion. Total RNA (20 μg) was used. Cyclophilin mRNA is shown as an estimation of the relative RNA loaded. D and E, Real-time PCR quantification of neomycin (D) and *Dio3* (E) mRNA in several tissues of *Dio3*^*Del m*−/*p*+^ and *Dio3*^*Del m*+/*p*−^ mice. Tissues include E16.5 fetuses (excluding the head and the liver), E16.5 liver and brain, P90 brain, and P5 testes. Data are represented as a percentage of expression from the paternal allele. Data represent the mean ± SEM of determinations in six (neomycin mRNA) or three to five (Dio3 mRNA) animals per group. *, *P* < .01 as determined by the Student's *t* test.

We have generated by homologous recombination in mouse ES cells an additional mouse model in which an approximately 4.5-kb genomic fragment between endogenous *Not*I and *Hin*dIII restriction sites has been deleted in the *Dio3* locus and replaced by the neomycin cassette used for clone selection. The deleted fragment contains the *Dio3* exon, 1.8 kb of 3′-flanking region and approximately 500 bp of ′-flanking region including the *Dio3* promoter (Figure 8A). A Southern blot analysis of *Eco*RI-digested gDNA isolated from tail snips of wild-type mice and mice heterozygous and homozygous for the deletion demonstrated the germline transmission of the deletion (Figure 8B). A Northern blot analysis of RNA isolated from a pregnant uterus and fetal heads demonstrated the absence of *Dio3* mRNA in mice homozygous for the deletion (*Dio3* Del −/−) (Figure 8C). Using this animal model, we analyzed mRNA expression of neomycin and *Dio3* in various tissues of heterozygous animals with different parental inheritance of the deletion. In the fetal liver, brain, and body of the fetus, neomycin mRNA levels were much higher in animals with paternal inheritance of the deletion (*Dio3* Del m+/p−) than in heterozygous animals that inherited the deletion from the mother (*Dio3* Del m−/p+) (Figure 8D). Because neomycin mRNA is expressed only from the deleted *Dio3* allele, these results indicate preferential expression from the paternal allele in these tissues.

In contrast, the contribution to neomycin mRNA levels was not significantly different between alleles in the neonatal testes and the adult brain (Figure 8D). Essentially the opposite pattern is observed for *Dio3* mRNA levels. Fetal tissue *Dio3* mRNA levels were much lower in animals with paternal inheritance of the deletion (*Dio3* Del m+/p−) than in heterozygous animals that inherited the deletion from the mother (*Dio3* Del m−/p+) (Figure 8E). Again, contributions to the *Dio3* mRNA levels in the adult brain were similar between alleles (Figure 8E). These tissue-specific imprinting patterns of neomycin and *Dio3* mRNAs in this animal model recapitulate the results obtained in the *Dio3* mutation mouse model, in which the *Dio3* gene and promoter are intact. These results demonstrate that, at least in the tissues studied, the *Dio3* promoter is not necessary for conferring tissue specificity to the allelic expression at the *Dio3* locus. This is consistent with studies indicating that imprinting control is mediated by differentially methylated regions such as the IG-DMR at the *Dlk1-Meg3* part of the cluster. It also shows that the normal imprinting pattern can be imposed on the regulation of the phosphoglycerate kinase promoter driving the neomycin gene; therefore, specific characteristics of the *Dio3* promoter do not confer sensitivity to imprinting.

## Discussion

In mice, the absence of a functional D3 enzyme leads to systemic overexposure to T_3_ during development (8) and to excessive TH action in certain adult tissues, such as the CNS (14). Given the pleiotropic actions of TH, this overexposure leads to a number of abnormalities. Because *Dio3* is expressed from the paternal allele during fetal life (16, 17), it is reasonable to expect that heterozygous animals inheriting the *Dio3* mutation from their fathers (*Dio3* m+/p−) would feature similar abnormalities. We show herein that this is at least partly the case because *Dio3* m+/p− mice exhibit elevated serum T_3_ and suppressed serum T_4_ as neonates, growth retardation, and small testes. These findings are also observed in the *Dio3* null mouse, but their severity is significantly reduced in the *Dio3* m+/p− mutant. Furthermore, viability and fertility of *Dio3* m+/p− mice is significantly improved when compared with *Dio3*^−/−^ mice, and their serum TH levels appear normal in adult life (10). The more moderate phenotype observed in *Dio3* m+/p− mice compared with *Dio3*^−/−^ mice is likely due to considerable *Dio3* expression from the maternal allele in certain tissues.

We demonstrate that mouse ES cell clones targeted for *Dio3* inactivation exhibit a high degree of monoallelic *Dio3* expression at the mRNA and protein levels, suggesting that strong genomic imprinting of *Dio3* is present around the blastocyst stage of early development. This is not surprising because most imprinted genes, including those in the *Dlk1-Dio3* imprinted domain, manifest such patterned expression in ES cell lines and at early developmental stages (38). It is remarkable that all five *Dio3* targeted clones exhibited markedly deficient levels of *Dio3* expression, suggesting a significant bias in the homologous recombination event, which favored the preferentially expressed, presumably paternal allele. This suggests that the expression and associated alterations in chromatin facilitate homologous recombination. The result is also consistent with observations that suggest the existence of epigenetic boundaries that are associated with a differential timing for DNA replication at imprinted loci (39). If this is also the case for the *Dio3* locus, it is then possible that gene targeting is facilitated when the preferentially expressed allele is replicated.

Although marked preferential *Dio3* expression from the paternal allele is found in ES cells and the fetus as a whole, it does not necessarily apply to all tissues and cell types. Many imprinted genes exhibit tissue-specific patterns of allelic expression (40, 41). We show that in the fetal eye, both alleles contribute equally to *Dio3* expression. The absence of *Dio3* imprinting in this tissue suggests that deficient expression from the paternal *Dio3* allele only is less likely to have an impact on the retinal and vision phenotype that is observed in *Dio3* null mice (42). The absence of *Dio3* imprinting is also observed in the newborn testes. In this regard, the decrease in testis size in *Dio3* p−/m+ mice is much less severe than in *Dio3*^−/−^ mice (unpublished results, Hernandez, A.) and may be due to the still significant neonatal exposure to elevated circulating levels of T_3_ in these animals.

Other fetal tissues (eg, nonneural craniofacial tissue) show a high degree of preferential expression from the paternal allele, but it is the CNS that displays a wide regional variation in the relative contributions from each allele to overall *Dio3* expression. Except for the cerebellum, which exhibits equal *Dio3* expression from both alleles, most regions of the perinatal and late neonatal brain exhibit preferential *Dio3* expression from the paternal allele. However, *Dio3* genomic imprinting appears relaxed in some brain regions because the expression from the paternal allele alone is not sufficient to achieve normal *Dio3* expression levels, and the expression from the maternal allele is close to 50% of wild-type values in some cases. Only the perinatal hypothalamus exhibits an allelic expression pattern that is consistent with robust genomic imprinting of *Dio3*, but even in this brain region, the monoallelic pattern of expression is relaxed by weaning age. The overall relaxation of genomic imprinting of *Dio3* by weaning age in most brain regions is consistent with the reported late neonatal loss of imprinting of *Dlk1* in neurogenic structures of the brain (43) and the increased plasticity of brain imprinting for several genes in the cluster (37). Thus, it is possible that common mechanisms of imprinting loss or relaxation exist for the *Dlk1-Dio3* imprinted domain in the CNS and other tissues.

Different epigenetic mechanisms regulating the expression of the larger, approximately 2.7-kb *Dio3* mRNA transcript, expressed in the mouse brain, may also explain the relaxation of *Dio3* imprinting in this tissue. In the fetal brain, this transcript is as abundant as the better characterized 2.2-kb transcript but becomes by far the most abundant by P7, being the only *Dio3* transcript detected in the P7 neocortex by Northern analysis. In the untreated adult rodent brain, this transcript is also the most abundant (7). Indirect evidence from the studies of *DIO3* in human cell lines (44) and preliminary observations in our laboratory suggests that the larger *Dio3* mRNA contains additional sequence in the 5′-untranslated region of the mRNA, with transcription thus being directed by an unidentified upstream promoter. It is thus possible, as observed with other imprinted genes (41), that this alternative promoter may not be regulated by the same epigenetic mechanisms. At a more specific cell and tissue level, it is possible that other imprinting patterns of *Dio3* expression exist. In this regard, Sittig et al (45) reported modest preferential *Dio3* expression from the maternal allele in the hippocampus of a rat model of developmental alcohol exposure, whereas Correa et al (46) reported preferential *Dio3* expression from the maternal allele in pancreatic β-cells.

The varied tissue and developmental patterns of *Dio3* allelic expression suggest the existence of tissue-specific mechanisms of epigenetic regulation of *Dio3* expression. An analysis of the methylation status of two regions in the imprinted domain with known regulatory roles on allelic *Dio3* expression (36), the IG-DMR and the *Meg3* intron, reveal subtle differences in the degree of methylation between some tissues. Although statistically significant, these differences are rather small and may not explain the allelic expression observed in a given tissue. For instance, *Dio3* is not imprinted in the neonatal testes and cerebellum. These tissues show increased Meg3 intron methylation compared with fetal craniofacial tissue, in which *Dio3* is imprinted. In this case, the difference in methylation is associated with a difference in the ratio of allelic expression. However, *Meg3* intron methylation in the newborn hypothalamus, in which *Dio3* imprinting is robust, is similar to that in testes and cerebellum, which show no *Dio3* imprinting. It is thus uncertain whether these methylation differences influence the observed tissue specific pattern of allelic *Dio3* expression.

Studies on a new mouse model in which the *Dio3* gene and its characterized promoter are deleted and substituted by the neomycin gene driven by a different putative promoter reveal no significant role for the endogenous *Dio3* promoter in mediating tissue-specific *Dio3* imprinting. At least for the tissues analyzed in this mouse model, the patterns of neomycin and *Dio3* allelic expression resemble those observed for the intact and mutated allele in mice carrying the *Dio3* mutation. This demonstrates that sequences in the endogenous *Dio3* promoter are not relevant in mediating the tissue specificity of *Dio3* allelic expression. Although transcription of the antisense *Dio3os* gene may be affected in mice carrying this deletion, our results indicate that this mouse model can be a useful tool for imprinting studies and for other types of investigations that benefit from the absence of the *Dio3* mRNA and protein.

Overall, our observations indicate that genomic imprinting of *Dio3* varies significantly between tissues, developmental stages, and *Dio3* transcripts. This applies in particular to the brain, an organ in which *Dio3* is prominently expressed throughout life. It is possible that the extent of control conferred by the IG-DMR may differ between one tissue and another; because *Dio3* is the most distant from the IG-DMR end of the cluster, it may be more prone to distance effects, resulting in the loss of *Dio3* imprinting in some contexts. This likely explains the less severe gross phenotype of heterozygous mice with inactivation of the paternal *Dio3* allele (as compared with null mice) and suggests that certain abnormalities of the *Dio3* null mice will be more likely to occur than others when imprinting of *Dio3*, and thus *Dio3* dosage, is disrupted. In that context, it is likely that phenotypes that originate in tissues with high *Dio3* expression and a marked pattern of monoallelic expression will be the most susceptible to abnormal epigenetic regulation of *Dio3*. Future studies will address this hypothesis.

## Additional material

Supplementary data supplied by authors.
