# Supplementary material for: Genomic Imprinting Variations in the Mouse Type 3 Deiodinase Gene Between Tissues and Brain Regions
Source: Mol Endocrinol. 2014 Sep 18;28(11):1875–86. doi: 10.1210/me.2014-1210 (PMC4213365; doi:10.1210/me.2014-1210)
Supplement: Supplementary file 1 [file me-14-1210.pdf]

Supplemental Methods

Table 1. Sequence of primers used for genotyping and real time qPCR (5' to 3').

Forward	Reverse	Use
GGAGTCCTGCTGCTTTTGTG	CGAGCCTCTCTGCAATTCAG	*Dio3* expression and mutation genotype
CCAGCTCATTCCTCCACTATG	AGGACACTTCCCTCTTTCCCTG	*Dio3* deletion genotype
GGAGTCCTGCTGCTTTTGTG	CTTTCCCTATCTCGAGGGAC	*Dio3* mutated allele expression
AGGAGCGAGACCCCACTAAC	CGGAGATGATGACCCTTTTG	Gapdh expression
GATCTCGTCGTGACCCATG	CGTCAAGAAGGCGATAGAAG	Neomycin expression

Methylation Analysis information. Sequence of the amplicons and location of the CG groups analyzed in the bisulfite sequencing and pyrosequencing determinations.

Bisulfite Sequencing

IG-DMR: mm10, chr12: 109528065-109528526

Unconverted sequence

ATTTTATAGTACA**CG**CTATATTTGTGCTAAGGTACATCATGCTAGTGTTAGGAAGGATTG
TGAATCTATA**CG**GAGATGTGCTGTGGACCCAGGCTGCAGTTCA**CG**AT**CG**ACTAGTACA
CAGGCTGACCATGTACAAGTGCTGTGGTTTGTCATGGGCAAGTCCCATGGCTTACTGTA
CACAATGCTGC**CG**TT**CG**CTATGAACTAC**CG**CTA**CG**GTTCATAGTGGACAGTCAGTGC**C**
GCAGAT**CG**CTATGGACTGGTGCCAAGGTT**CG**CCATGGACTAGTGC**CGCG**GACCTC**CG**
TGAACTAG**CG**AGGAGGTT**CG**C**CG**TGTACTAATGC**CG**CTT**CGCG**TAC**CG**CTGTGTA**CGC**
GTGC**CGCG**AAC**CG**C**CG**TGGAATTGTGC**CGCG**GTT**CG**C**CG**TGGAGTAG**CG**CTGCAGC**C**
GCTATGCTATGCTGTTTCTTTCTTTTCCTTAACTCCTGGAGTGAGGGAAGGGCTG

Pyrosequencing

IG-DMR: mm10 chr12:109528205-109528523

Unconverted sequence

GTGGTTTGTCATGGGCAAGTCCCATGGCTTACTGTACACAATGCTGC**CG**TT**CG**CTATGA
ACTACCGCTA**CG**GTTCATAGTGGACAGTCAGTGC**CG**CAGAT**CG**CTATGGACTGGTGCC
AAGGTT**CG**CCATGGACTAGTGC**CG**CGGACCTCCGTGAACTAGCGAGGAGGTTCGCCG
TGTACTAATGCCGCTTCGCGTACCGCTGTGTACGCGTGCCGCGAACCGCCGTGGAATT
GTGCCGCGGTTCGCCGTGGAGTAGCGCTGCAGCCGCTATGCTATGCTGTTTCTTTCTT
TTCCTTAACTCCTGGAGTGAGGGAAGGG

Meg3 intron: mm10 chr12:109542982-109543085

Unconverted sequence

CCTGTGGGGGTGCCTCAGGTGGTTGGGCTATTGGAGTCTTAGTGAGTG**CG**GGC**CGCG**
CGCCCCCTAG**CG**GTCTCTATGTGCAAATGGTTCTTGGCCTGCCAGAC
